# Correction: An assessment of the value of deep neural networks in genetic risk prediction for surgically relevant outcomes

**DOI:** 10.1371/journal.pone.0346168

**Published:** 2026-03-30

**Authors:** The PLOS One Staff

The following information is missing from the Data Availability statement: Analytic methods and code are available from https://github.com/satihest/surgical_complications.

The publisher apologizes for the error.
