# Supplementary material for: An assessment of the value of deep neural networks in genetic risk prediction for surgically relevant outcomes
Source: PLoS One. 2024 Jul 15;19(7):e0294368. doi: 10.1371/journal.pone.0294368 (PMC11249253; doi:10.1371/journal.pone.0294368)
Supplement: S1 Table — (DOCX) [file pone.0294368.s001.docx]

**Supplementary table 1:** List of ICD-9 and ICD-10 codes used for the phenotypes in question.

| ICD-9 codes for atrial fibrillation | 4273 |
| --- | --- |
| ICD-10 codes for atrial fibrillation | I48, I480, I481, I483, I484, I489 |
| ICD-9 codes for venous thromboembolism | 4151, 4511, 4512, 4519, 4531, 4532, 4534, 4538, 4539, 4534, 4531, 4532, 4539 |
| ICD-10 codes for venous thromboembolism | I260, I269, I801, I802, I803, I808, I809, I820, I821, I822, I823, I828, I829, O082, O223, O871, O882, I81 |
| ICD-9 codes for pneumonia | 4810, 4820, 4821, 4823, 4824, 4828, 4829, 4830, 4831, 4838, 4840, 4841, 4843, 4835, 4836, 4847, 4848, 4850, 4860 |
| ICD-10 codes for pneumonia | J13, J14, J150, J151, J152, J153, J154, J155, J156, J157, J158, J159, J16, J160, J168, J170, J172, J173, J178, J180, J181, J182, J188, J189, J851 |
