# Supplementary material for: An assessment of the value of deep neural networks in genetic risk prediction for surgically relevant outcomes
Source: PLoS One. 2024 Jul 15;19(7):e0294368. doi: 10.1371/journal.pone.0294368 (PMC11249253; doi:10.1371/journal.pone.0294368)
Supplement: S2 Table — (DOCX) [file pone.0294368.s002.docx]

**Supplementary table 2:** List of OPCS-4 codes used to define surgery.

| ['W401', 'W371', 'W381', 'W822', 'Q074', 'M021', 'K453', 'J183', 'M611', 'G693', 'W201', 'W205', 'M025' , 'T202', 'T413', 'W852', 'W192', 'W411', 'H335', 'J692', 'W164', 'W191', 'T252', 'K262', 'W421', 'G753', 'W283', 'Q221', 'A054', 'S065', 'W373', 'W391', 'B282', 'W593', 'M022', 'W153', 'A401', 'H114', 'H333', 'H114', 'J021', 'V255', 'T309', 'H053', 'W199', 'W581', 'L841' , 'L931' , 'L948' , 'W242', 'A411', 'S472' , 'A651' , 'W461 ', 'L851' , 'S069' , 'C751' , 'W192' , 'W941' , 'W403' , 'L622' , 'W241' , 'E543' , 'T676', 'S571' , 'B274' , 'Q075' , 'H071' , 'H336' , 'R182' , 'G743' , 'A025' , 'W879' , 'T962' , 'G011' , 'H012' , 'W742' , 'G331' , 'W858' , 'J011' , 'T791' , 'H041' , 'H511' , 'W833' , 'M341' , 'L185' , 'W931' , 'A021' , 'H334' , 'T439' , 'B285' , 'A022' , 'W083' , 'W246' , 'H338' , 'W791' , 'B279' , 'G031' , 'W521' , 'W621' , 'W243' , 'T838' , 'L191' , 'J699' , 'T969', 'A013', 'W654', 'W085', 'A611', 'K403', 'O291', 'P232', 'H331', 'A671', 'Q013', 'K263', 'W042', 'H073', 'T391', 'L843', 'H011', 'W198', 'H095', 'L845', 'L192', 'E528', 'L516', 'G218', 'A023', 'T678', 'M012', 'M612', 'K402', 'L871', 'W571', 'V339', 'H074', 'A384', 'A388', 'W208', 'M538', 'W871', 'M278', 'W424', 'M421', 'V337', 'H072', 'G698', 'Q231', 'W152', 'V468', 'H042', 'T273', 'G243', 'B293', 'A295', 'G784', 'J576', 'B311', 'W222', 'T423', 'G699', 'L842', 'W802', 'R172', 'V254', 'W082', 'T272', 'W451', 'T521', 'L593', 'V253', 'V256', 'V209', 'P242', 'H103', 'W431', 'W382', 'S551', 'T723', 'V411', 'W471', 'W423', 'A389', 'B083', 'T748', 'M013', 'J023', 'T242', 'T679', 'V291', 'W301', 'H062', 'H051', 'J561', 'V221', 'S068', 'A383', 'Q089', 'W803', 'G648', 'G352', 'E032', 'E248', 'V031', 'G521', 'T331', 'J091', 'W122', 'G532', 'H092', 'W821', 'W402', 'F349', 'M039', 'F442', 'W961', 'H013', 'L591', 'J185', 'T162', 'G748', 'T531', 'B181', 'V462', 'W399', 'M231', 'B312', 'W384', 'V433', 'G694', 'L181', 'Q092', 'W951', 'A108', 'H105', 'W194', 'L258', 'V143', 'H079', 'E541', 'T316', 'H091', 'L601', 'J569', 'W712', 'G289', 'L541', 'W282', 'G303', 'E542', 'W211', 'Q079', 'M051', 'J552', 'K255', 'H332', 'W202', 'H151', 'S022', 'W752', 'A511', 'G028', 'L131', 'L233', 'S172', 'W158', 'R181', 'W743', 'A473', 'M014', 'V336', 'W288', 'H029', 'W212', 'T278', 'W891', 'V403', 'F222', 'H412', 'K334', 'A445', 'T551', 'G234', 'W859', 'V037', 'J582', 'J042', 'J571', 'K301', 'J024', 'J571', 'K301', 'J024', 'W195', 'L581', 'H131', 'G633', 'M373', 'N288', 'W834', 'V294', 'V039', 'V152', 'H052', 'M191', 'W393', 'L198', 'S251', 'K412', 'A412', 'T792', 'S552', 'H142', 'H064', 'V241', 'J012', 'G283', 'L343', 'F231', 'F451', 'W193', 'L844', 'H085', 'W308', 'W332', 'T972', 'L582', 'O182', 'T229', 'G492', 'T458', 'E294', 'K335', 'T262', 'Q518', 'J578', 'V392', 'E148', 'H113', 'L583', 'G242', 'L682', 'L621', 'T412', 'T342', 'L798', 'V104', 'J025', 'A652', 'T622', 'W214', 'W629', 'H104', 'G281', 'K454', 'T671', 'W395', 'T243', 'H101', 'E036', 'A028', 'H112', 'L201', 'T525', 'L624', 'W422', 'H118', 'V383', 'B223', 'L628', 'B012', 'W232', 'T251', 'J028', 'K451', 'G312', 'L124', 'H061', 'L818', 'T203', 'O181', 'K023', 'L602', 'G278', 'W144', 'V313', 'P251', 'N113', 'E192', 'V242', 'W033', 'C134', 'L193', 'G734', 'M348', 'F443', 'E042', 'B284', 'G601', 'W523', 'G789', 'G013', 'W832', 'J182', 'J219', 'L592', 'W196', 'T212', 'W068', 'M619', 'W433', 'T675', 'G362', 'W238', 'V402', 'G584', 'W131', 'T798', 'V259', 'T724', 'K253', 'H152', 'K021', 'W038', 'W231', 'V223', 'M359', 'T259', 'E532', 'T318', 'H019', 'T222', 'G232', 'W091', 'A386', 'H358', 'L194', 'J189', 'K272', 'W758', 'G251', 'T209', 'W372', 'L598', 'L587'] |
| --- |
