# Supplementary material for: An assessment of the value of deep neural networks in genetic risk prediction for surgically relevant outcomes
Source: PLoS One. 2024 Jul 15;19(7):e0294368. doi: 10.1371/journal.pone.0294368 (PMC11249253; doi:10.1371/journal.pone.0294368)
Supplement: S3 Table — (DOCX) [file pone.0294368.s003.docx]

**Supplementary table 3:** Demographics and comorbidities with ICD-codes

| Demographics | **Age** (UKB code field: 21022-0.0)  **Sex** (UKB code field: 31-0.0) |
| --- | --- |
| Comorbidities | **Previous or current smoker** (UKB code field: 1249-0.0, 1249-1.0, 1249-2.0, 1249-3.0)  **Hypertension** (ICD-10: I10, I100, I101, I102, I103, I104, I105, I106, I107, I108, I109, I13, I130, I131, I132, I133, I134, I135, I136, I137, I138, I139, I15, I150, I151, I152, I153, I154, I155, I156, I157, I158, I159)  **Cancer** (UKB code field: 2453-0.0, 2453-1.0, 2453-2.0, 2453-3.0 ), heart failure (ICD-10: I50, I501, I502, I5020, I5021, I5022, I5023, I503, I5030, I5031, I5032, I5033, I504, I5041, I5042, I5043, I508, I5081, I50810, I50810, I50811, I50812, I50813, I50814, I5082, I5083, I5084, I5089, I509)  **BMI** (UKB code field: 21001-0.0, 21001-1.0, 21001-2.0, 21001-3.0. |
