# Supplementary material for: An assessment of the value of deep neural networks in genetic risk prediction for surgically relevant outcomes
Source: PLoS One. 2024 Jul 15;19(7):e0294368. doi: 10.1371/journal.pone.0294368 (PMC11249253; doi:10.1371/journal.pone.0294368)
Supplement: Attachment — Submitted filename: Response_to_reviewers.docx [file pone.0294368.s004.docx]

Blegdamsvej 9

# 2100 Copenhagen Denmark

# Dear Dr. Zhu and reviewers,

We thank you for reading our manuscript and we are pleased that you consider it for publication in PLOS One.

We have responded to the reviewer’s comments below and adjusted the manuscript accordingly. We believe the manuscript has improved and would like to thank the reviewers and editor for this.

The changes made to the manuscript are highlighted with red inside the manuscript and in this letter. Deleted paragraphs are highlighted with ~~red strikethrough~~. We have furthermore attached a version of the revised manuscript with the changes in normal, black fond.

We are looking forward to receiving your response,

Respectfully,

Martin Sillesen, MD, PhD, Associate Professor of Surgery

**Editorial comments:**

*1. Please ensure that your manuscript meets PLOS ONE's style requirements, including those for file naming. The PLOS ONE style templates can be found at* 
[*https://journals.plos.org/plosone/s/file?id=wjVg/PLOSOne_formatting_sample_main_body.pdf*](https://journals.plos.org/plosone/s/file?id=wjVg/PLOSOne_formatting_sample_main_body.pdf) *and* 
[*https://journals.plos.org/plosone/s/file?id=ba62/PLOSOne_formatting_sample_title_authors_affiliations.pdf*](https://journals.plos.org/plosone/s/file?id=ba62/PLOSOne_formatting_sample_title_authors_affiliations.pdf)

**Answer:**

Thank you. We have updated the manuscript, figures and tables accordingly.

*2. Please note that PLOS ONE has specific guidelines on code sharing for submissions in which author-generated code underpins the findings in the manuscript. In these cases, all author-generated code must be made available without restrictions upon publication of the work. Please review our guidelines at [https://journals.plos.org/plosone/s/materials-and-software-sharing#loc-sharing-code](https://journals.plos.org/plosone/s/materials-and-software-sharing#loc-sharing-code) and ensure that your code is shared in a way that follows best practice and facilitates reproducibility and reuse.*

**Answer:**

We have now made all queries and code available at <https://github.com/satihest/surgical_complications>.

*3. In the online submission form, you indicated that data is not publicly available but can be applied for at [https://www.ukbiobank.ac.uk/enable-your-research/apply-for-access](https://www.ukbiobank.ac.uk/enable-your-research/apply-for-access). Analytic methods will be made public at github.com at request. Requests to access these datasets should be directed to [https://www.ukbiobank.ac.uk/enable-your-research/apply-for-access](https://www.ukbiobank.ac.uk/enable-your-research/apply-for-access).
All PLOS journals now require all data underlying the findings described in their manuscript to be freely available to other researchers, either 1. In a public repository, 2. Within the manuscript itself, or 3. Uploaded as supplementary information.
This policy applies to all data except where public deposition would breach compliance with the protocol approved by your research ethics board. If your data cannot be made publicly available for ethical or legal reasons (e.g., public availability would compromise patient privacy), please explain your reasons on resubmission and your exemption request will be escalated for approval.*

**Answer:**

Thank you. We have included a statement below:

The de-identified dataset used for this study can be obtained from the authors, provided written authorization from UK Biobank can be obtained. Authors are not allowed to share data without express permission from this governing body. The UK Biobank can be contacted at <https://www.ukbiobank.ac.uk/learn-more-about-uk-biobank/contact-us>. Enquiries from researchers about applying for access should be directed to the Access Management Team – email: [access@ukbiobank.ac.uk](mailto:access@ukbiobank.ac.uk).

*4. Please amend your manuscript to include your abstract after the title page.*

**Answer:**

We have now invluded the abstract after the title page.

*5. We notice that your supplementary [figures/tables] are included in the manuscript file. Please remove them and upload them with the file type 'Supporting Information'. Please ensure that each Supporting Information file has a legend listed in the manuscript after the references list.*

*Additional Editor Comments :

In addition to address the critical comments from both Reviewers in full, I would encourage that the authors follow the latest version of the Polygenic Risk Score Reporting Standards (PMID: 33692554) to ensure the methodological transparency and scientific rigor in the revised manuscript.*

**Answer:**

We have now deleted the supplementary material from the manuscript and included only titles/legends after references. Further, we have updated the manuscript accordingly with the Polygenic Score Reporting Standards.

**Reviewer 1**:

**R-1-1**:

*The paper is interesting only one problem is the language. I suggest major revision of the English. The abstract, introduction, material and methods, discussion and conclusions are good structured and with interesting results.*

**Answer**:

We would like to thank the reviewer for taking the time to review our paper. We are happy that the reviewer found our paper interesting, and we appreciate the relevant feedback on the language. Accordingly, we have had the paper reviewed by an American physician scientist (Dr. Jaimie Chang, MD, resident physician at Rush Medical Center) and have updated the grammar and language accordingly to her suggestions. Please see the entire revised manuscript for the updates.

**Reviewer 2**:

*Predicting surgically relevant outcomes is an important clinical problem to make better curation on the patient. Christensen et al compare several machine learning approaches that can predict patients’ surgically relevant outcomes including atrial fibrillation, venous thromboembolism, and pneumonia. They compared the performance by using different combinatorial features (SNPs/clinical information/both) and models (linear model, deep neural networks). Based on the prediction result, the author claimed that the deep neural network out-performs linear model. However, in my experience, I have many concerns.*

**R-2-1**:

*The author does not provide the script and code of the work. I suggest the author provide the code and the dependency for reproducibility. I also suggest author provide the preprocessed data for training model.*

**Answer**:

First of all, we would like to thank the reviewer for taking the time to review our paper and for the relevant critique. We believe the suggestions have improved the manuscript. We agree that the code should be publicly available, and have therefore uploaded all Python code, shell scripts and UK Biobank queries used on the following Github account: <https://github.com/satihest/surgical_complications>

Unfortunately, UK Biobank data is not publicly available, but can be applied for by anyone. We cannot, without their permission, upload the unprocessed training data.

**R-2-2**:

*The feature selection process is not correct. In the machine learning process, the dataset should be split into a training set and a test set at the beginning. Based on the training set, the author can perform feature selection (SNP selection). The test set should be set aside until final testing to get the performance of the model. Otherwise, the information in the test set will be leaked to the model, which make the performance of the model overestimated. However, the author select the top 100 SNPs based on the whole dataset including training and test set. I think this might be the reason for the great AUC performance of the linear model. The performance of DNN seems not as good as the linear model.*

**Answer**:

We agree, and we have updated the analyses accordingly. Please see the revised manuscript for the updated results. Overall, there were no major changes in the SNPs generated from doing the GWAS models on only the training data, and the models were therefore comparable in predictability. However, there were changes in the SNPs most important for the models.

Please see below and in the revised results and discussion in the revised manuscript.

*Changes to the manuscript, Page (P) 8, line (l) 19:*

The cohorts were split into training/validation and test sets before, and only the training data was used for the initial GWAS-models. Relevant GWAS plots, including Manhattan and Quantile-Quantile (QQ) plots were generated using qqman

*Changes to the manuscript, P 10, l 6:*

Baseline characteristics are listed in table 1. The SNP model reached a ROC-AUC of ~~60.9% [95% CI, 60.6%-61.0%]~~ 60.3 [95% CI, 60.0%-60.4%]. All individuals were classified as not having AF. The PRAUC was 0.09. The clinical model reached a ROC-AUC of 78.7% [95% CI, 78.7%-78.7%] with a recall of 9% and a precision of 53%. The PRAUC was 0.25. The combined model reached a ROC-AUC of ~~80.1% [95% CI, 80.0%-80.1%] with a recall of 9% and a precision of 57%~~ 80.0% [95% CI, 79.9%-80.0%] with a recall of 9% and a precision of 56%. The PRAUC was 0.28.

*Changes to the manuscript, P 12, l 6:*

Baseline characteristics for VTE are listed in table 2. The SNP model reached a ROC-AUC of ~~59.6% [95% CI, 59.0%-59.7%]~~ 60.1% [95% CI, 59.6%-60.4%]. All individuals were classified as not having VTE. The PRAUC was 0.04. The clinical model reached a ROC-AUC of 63.4% [95% CI, 63.2%-63.4%]. All individuals were classified as not having VTE. The PRAUC was 0.04. The combined model reached a ROC-AUC of ~~66.1% [95% CI, 65.7%-66.1%]~~ 66.6% [95% CI, ]. All individuals were classified as not having VTE. The PRAUC was 0.05

*Changes to the manuscript, P 14, l 5:*

Baseline characteristics are listed in table 3. The SNP model reached a ROC-AUC of ~~57.3% [95% CI, 56.5%-57.4%]~~ 50.1% [95% CI, 49.6%-50.6%]. All individuals were classified as not having pneumonia. The PRAUC was 0.04. The clinical model reached a ROC-AUC of 69.2% [95% CI, 69.1%-69.2%]. All individuals were classified as not having pneumonia. The PRAUC was 0.12. The combined model reached a ROC-AUC of ~~70.5% [95% CI, 70.2%-70.6%] with a recall of 0.01% and a precision of 0.4%~~ 68.4% [95% CI, 68.0%-68.5%] with a recall of 0.01 and a precision of 0.5. The PRAUC was 0.11.

*Changes to the manuscript, P 16, l 7:*

The SNP model reached a ROC-AUC of ~~59.9% [95% CI, 58.6%-61.3%]~~ 59.4% [95% CI, 58.2%-60.9%]in the test set. Recall was ~~36.9%~~ 42.8% and precision was ~~9.3%~~ 8.7%, with the area under the precision-recall curve (PRAUC) being 0.09. The clinical model reached a ROC-AUC of 78.8% [95% CI, 77.8%-79.8%] with recall and precision of 72.0% and 13.5%, respectively, with the PRAUC being 0.25. The combined model reached a ROC-AUC of ~~79.4% [95% CI, 78.8%-80.5%]~~ 79.8 [95% CI, 78.8%-80.9%] with a recall and precision of 74.8% and 13.5%, respectively, with the PRAUC being 0.27.

*Changes to the manuscript, P 18, l 4:*

The SNP model reached a ROC-AUC of ~~60.0% [95% CI, 57.8%-61.8%]~~ 51.5% [95% CI, 49.4%-53.4%] with a recall of 50.8% and precision of 4% and a PRAUC of 0.03. The clinical model reached a ROC-AUC of 63.2% [95% CI, 61.2%-65.0%] with a recall and precision of 67.5% and 4.0%, respectively, and a PRAUC of 0.05. The combined model reached a ROC-AUC of ~~65.4% [95% CI, 63.6%-67.2%]~~ 62.6% [95% CI, 60.7%-64.5%] with a recall and precision 68.8% and 4.0%, respectively, and a PRROC of 0.05

*Changes to the manuscript, P 19, l 5:*

The SNP model reached a ROC-AUC of ~~55.5% [95% CI, 54.1%-56.9%]~~ 51.0% [95% CI, 49.7%-52.4%] with a recall of 55.0% and precision of 5% and a PRAUC of 0.05. The clinical model reached a ROC-AUC of 69.7% [95% CI, 68.5%-70.8%] with a recall and precision of 67.7% and 7.4%, respectively and a PRAUC of 0.13. The combined model reached a ROC-AUC of ~~69.9% [95% CI, 68.7%-71.0%]~~ 69.7% [95% CI, 68.6%-70.8%] with a recall and precision of 70.1% and 7.3%, respectively, and a PRAUC of 0.12.

*Changes to the manuscript, P 21, l 6:*

Overall, we found that adding SNP data to clinical risk prediction models enhanced the predictive power~~, and that the GLN approach seemed superior to a legacy linear risk prediction approach.~~

*Changes to the manuscript, P 21, l 15:*

The GLN-models ~~performed better on recall and precision, and it~~ ~~was~~ were able to classify positives correctly with just genomic information, however, the PRAUC was generally comparable between the deep learning and linear models with identical data. Given that the linear and GLN models utilize distinct tuning parameters for hard predictions, a direct comparison of recall and precision may not be critically significant, although the deep learning models generally performed better. However, as hard predictions are necessary in a clinical setting, a discussion is still warranted. Precision-recall curves are depicted in supplementary materials.

*Changes to the manuscript, P 21, l 22:*

It is exemplified for AF, where the genetic linear model had a ROC-AUC of ~~60.9% [95% CI 69.6%-61.0%] while the GLN had a ROC-AUC of 59.9% [95% CI 5.8.6%-61.3%]~~ 60.3% [95% CI, 60.0%-60.4%] while the GLN had a ROC-AUC of 59.4% [95% CI, 58.2%-60.9%]. The recall, however, was 0% and ~~36.9%~~ 42.8%,

*Changes to the manuscript, P 22, l 2:*

On the contrary, the GLN-model had a recall of ~~36.9%~~ 42.8% and therefore identifies ~~around~~ more than one third of cases correctly, which heightens the likelihood of clinical meaningful utilization considering only SNPs were included in the model. However, the precision was calculated low at ~~4%,~~ 8.7%, which would lead to overdiagnosis and possibly overtreatment.

*Changes to the manuscript, P 22, l 9:*

It, however, cannot be ruled out that the differences between the models are not due to an inherent predictive advantage in the GLN-model, but simply due to different hyperparameter tuning, as indicated by the similar performance in PRAUC.

*Changes to the manuscript, P 22, l 21:*

administrative codes are used for PC curation.(19)

~~GLN based models did, however, outperform linear approaches in terms of recall performance, might indicate that the ability to capture the effects of non-linear genetic traits on the overall phenotype, may be possible through this modelling approach~~.

*Changes to the manuscript, P 23, l 4:*

*Identified Single Nucleotide Polymorphisms*

~~In the GLN-model, rs3807989 was the most activated SNP in regards of classifying individuals with AF. It is an intron variant in~~ *~~CAV1~~* ~~which codes for a main component in caveolae plasma membrane and further acts as a tumor suppressor.(20, 21) It has been associated with a large variety of diseases including AF in numerous populations.(22, 23) Interestingly, the prevalence of the reference and risk allele is roughly equal which suggests the possibility of a relatively new mutation or that the risk variant has a different functional advantage which balances the selection~~. In the GLN model, rs17042171 was the most activated SNP in regards of classifying individuals with AF. It is an intergenic variant downstream from *PITX2* which codes for the paired-like homeodomain transcription factor 2.(24, 25) The risk allele is moderately prevalent in the European population with a prevalence of 15%. suggesting a negative selection pressure of the risk allele (26)

*Changes to the manuscript, P 23, l 16:*

a variant near 4q25, which has been extensively associated with AF in a variety of populations and is also in close proximity to *PITX2*.(25, 27), The variants most highly associated with AF in the linear model was also rs17042171~~, also a variant near 4q25~~. ~~The alternative allele has worldwide prevalence of up to 16% and 13% in the European population, which makes the risk variant very common, although not equal to the reference allele suggesting a negative selection pressure of the risk allele.(26)~~

*Changes to the manuscript, P 23, l 25:*

Besides the top variant, rs17042171, ~~T~~the difference in which variants show importance for the GLN and linear model, respectively, and that the GLN-models in general performed significantly better in recall compared with the matching linear, shows that non-linear interactions between genes which are potentially of great importance in the risk of a particular trait

*Changes to the manuscript, P 24, l 8:*

the genes have previously been described to be in a direct pathway or in any kind of interaction. ~~Interestingly, Gao et al. showed that the level of caveolin-1 determines the level of product of~~ *~~KCNN1~~* ~~which previously has been highly associated with AF in several GWAS-studies.(22, 28-30)~~

*Changes to the manuscript, P 24, l 11:*

The SNPs with highest importance for classifying VTE in the GLN-model was ~~rs505922~~ rs8176745, a synonymous variant in, ~~an intron variant~~ in *ABO*.(31) The ~~variant has previously been associated~~ variant has, to our knowledge, not previously been associated with VTE.(6)

*Changes to the manuscript, P 24, l 19:*

The SNP that had the highest feature importance in the GLN-model for classifying bacterial pneumonia was ~~rs11080143~~ rs17851582 for the SNP-model and rs9353801 for the combined model

*Changes to the manuscript, P 24, l 22:*

but rather due to chance alone. rs17851582 is a missense variant in *GAMT* which codes for the liver enzyme guanidinoacetate methyltransferase. Deficiency of the enzyme can affect brain and muscle development and lead to severe neurological problems including epilepsy.(32) However, the specific variant in our analysis is deemed to be benign.(33) rs9353801 was most important in the combined model. It has no reported clinical significance in the literature and does not lie close to any biological meaningful genes. One downstream gene, *CASC6*, codes for cancer susceptibility 6, which, although the name may indicate otherwise, has an unknown function.. ~~This sentiment is further supported by the fact that rs11080143 has no reported clinical significance in the literature and does not lie close to any biological meaningful genes~~. ~~One downstream gene,~~ *~~KSR1~~*~~, has been associated with different malignancies including breast adenocarcinoma and thyroid cancer.(34, 35) To our knowledge,~~ *~~KSR1~~* ~~has not been associated directly with lung cancer, but considering the protein product has a positive downstream signaling function of the RAS/MAPK pathway, and the association with other cancers, a connection seems likely~~.

*Changes to the manuscript, P 25, l 14:*

In the linear model, rs10519203 was the most associated with classifying pneumonia in the combined model and second most associated in the genetic model.

**R-2-3**:

*The performance of the model should be measured by AUC rather than a recall value that depends on a specific cutoff. To measure the imbalance data, I suggest authors use the area under the precision-recall curve (PRAUC) instead, which is more sensitive to imbalance data. AUC and PRAUC can measure the global performance of machine learning better.*

**Answer**:

Thank you for this suggestion, and we agree. We have now included the PRAUC in the results. Please find the changes below and in the revised manuscript. We have further deleted all heatmap ROC-AUC development figures, as they do not provide any additional or supporting information.

*Changes to the manuscript, P 8 l 22:*

Performance plots including the Receiver Operator Characteristics of the Area Under the Curve (ROC-AUCs), Area Under the Precision-Recall Curve (PRAUC) and heatmaps were created using Scikit-learn 1.2.1 (Python 3).(15)

*Changes to the manuscript, P 10 l 17:*

***Linear models***

*Atrial fibrillation*

Baseline characteristics are listed in table 1. The SNP model reached a ROC-AUC of ~~60.9% [95% CI, 60.6%-61.0%]~~ 60.3 [95% CI, 60.0%-60.4%]. All individuals were classified as not having AF. The PRAUC was 0.09. The clinical model reached a ROC-AUC of 78.7% [95% CI, 78.7%-78.7%] with a recall of 9% and a precision of 53%. The PRAUC was 0.25. The combined model reached a ROC-AUC of ~~80.1% [95% CI, 80.0%-80.1%] with a recall of 9% and a precision of 57%~~ 80.0% [95% CI, 79.9%-80.0%] with a recall of 9% and a precision of 56%. The PRAUC was 0.28.

*Changes to the manuscript, P 12 l 6:*

*Venous thromboembolism*

Baseline characteristics for VTE are listed in table 2. The SNP model reached a ROC-AUC of ~~59.6% [95% CI, 59.0%-59.7%]~~ 60.1% [95% CI, 59.6%-60.4%]. All individuals were classified as not having VTE. The PRAUC was 0.04. The clinical model reached a ROC-AUC of 63.4% [95% CI, 63.2%-63.4%]. All individuals were classified as not having VTE. The PRAUC was 0.04. The combined model reached a ROC-AUC of ~~66.1% [95% CI, 65.7%-66.1%]~~ 66.6% [95% CI, ]. All individuals were classified as not having VTE. The PRAUC was 0.05.

*Changes to the manuscript, P 14 l 6:*

*Pneumonia*

Baseline characteristics are listed in table 3. The SNP model reached a ROC-AUC of ~~57.3% [95% CI, 56.5%-57.4%]~~ 50.1% [95% CI, 49.6%-50.6%]. All individuals were classified as not having pneumonia. The PRAUC was 0.04. The clinical model reached a ROC-AUC of 69.2% [95% CI, 69.1%-69.2%]. All individuals were classified as not having pneumonia. The PRAUC was 0.12. The combined model reached a ROC-AUC of ~~70.5% [95% CI, 70.2%-70.6%] with a recall of 0.01% and a precision of 0.4%~~ 68.4% [95% CI, 68.0%-68.5%] with a recall of 0.01 and a precision of 0.5. The PRAUC was 0.11.

*Changes to the manuscript, P 16 l 7:*

***Deep learning models***

*Atrial fibrillation*

The SNP model reached a ROC-AUC of ~~59.9% [95% CI, 58.6%-61.3%]~~ 59.4% [95% CI, 58.2%-60.9%]in the test set. Recall was ~~36.9%~~ 42.8% and precision was ~~9.3%~~ 8.7%, with the area under the precision-recall curve (PRAUC) being 0.09. The clinical model reached a ROC-AUC of 78.8% [95% CI, 77.8%-79.8%] with recall and precision of 72.0% and 13.5%, respectively, with the PRAUC being 0.25. The combined model reached a ROC-AUC of ~~79.4% [95% CI, 78.8%-80.5%]~~ 79.8 [95% CI, 78.8%-80.9%] with a recall and precision of 74.8% and 13.5%, respectively, with the PRAUC being 0.27

*Changes to the manuscript, P 18 l 4:*

*VTE*

The SNP model reached a ROC-AUC of ~~60.0% [95% CI, 57.8%-61.8%]~~ 51.5% [95% CI, 49.4%-53.4%] with a recall of 50.8% and precision of 4% and a PRAUC of 0.03. The clinical model reached a ROC-AUC of 63.2% [95% CI, 61.2%-65.0%] with a recall and precision of 67.5% and 4.0%, respectively, and a PRAUC of 0.05. The combined model reached a ROC-AUC of ~~65.4% [95% CI, 63.6%-67.2%]~~ 62.6% [95% CI, 60.7%-64.5%] with a recall and precision 68.8% and 4.0%, respectively, and a PRROC of 0.05

*Changes to the manuscript, P 19 l 5:*

*Pneumonia*

The SNP model reached a ROC-AUC of ~~55.5% [95% CI, 54.1%-56.9%]~~ 51.0% [95% CI, 49.7%-52.4%] with a recall of 55.0% and precision of 5% and a PRAUC of 0.05. The clinical model reached a ROC-AUC of 69.7% [95% CI, 68.5%-70.8%] with a recall and precision of 67.7% and 7.4%, respectively and a PRAUC of 0.13. The combined model reached a ROC-AUC of ~~69.9% [95% CI, 68.7%-71.0%]~~ 69.7% [95% CI, 68.6%-70.8%] with a recall and precision of 70.1% and 7.3%, respectively, and a PRAUC of 0.12

**R-2-4**:

*In the method session, the top feature selection process of 100 SNPs is merged into the session of the linear model, which makes the reader confused about whether the DNN uses all features or these selected features.*

**Answer**:

Thank you for noticing this. The headlines were indeed wrong, and we have changed it accordingly. Please find the changes below and in the revised manuscript.

*Changes to the manuscript, P 8 line 8:*

*~~Linear Polygenic risk score (PGS) modelling approach~~*

*GWAS*

The initial GWAS-analyses were analyzed using a mixed linear model (MLM)

*Changes to the manuscript, P 9 line 1:*

*Linear Polygenic risk score (PGS) modelling approach*

A linear PGS was generated using the logistic regression module as implemented in scikit-learn 1.2.1 for Python 3

**R-2-5**:

*The author only provides the top feature of the model. I suggest the authors provide the information of positive or negative effects (coefficient and SHAP value) to the prediction value. Ensuring the supporting literature matches the effect is necessary.*

**Answer**:

The positive and negative coefficients have now been added to the relevant tables. Please find the change below and in the revised manuscript:

*Changes to the manuscript, P 11 l 2:*

**Table 4B:** Table of the SNPs with the highest feature importance for the genetic, and mixed atrial fibrillation linear models

| **Genetic, SNP** | **Coefficient (positive prediction)** | **Gene** | **Mixed, SNP** | **Coefficient (positive prediction)** | **Gene** |
| --- | --- | --- | --- | --- | --- |
| rs17042171 | 0.282965 | Intergenic | rs17042171 | 0.341726 | Intergenic |
| rs10033464 | 0.218606 | Intergenic | rs10033464 | 0.248286 | Intergenic |
| rs3731748 | 0.182317 | *TTN/TTN-AS1* | rs3731748 | 0.232892 | *TTN/TTN-AS1* |
| rs2723065 | 0.160737 | *LINC02576* | rs3829747 | 0.205116 | *TTN/TTN-AS1* |
| rs13390491 | 0.157113 | *TTN* | rs13390491 | 0.176328 | *TTN* |

*Changes to the manuscript, P 13 l 2:*

**Table 4D:** Table of the SNPs with the highest feature importance for the genetic, and mixed Venous thromboembolism linear models

| **Genetic, SNP** | **Coefficient (positive prediction)** | **Gene** | **Mixed, SNP** | **Coefficient (positive prediction)** | **Gene** |
| --- | --- | --- | --- | --- | --- |
| rs4524 | 0.329348 | *F5* | rs4524 | 0.304659 | *F5* |
| rs59262400 | 0.286520 | *TSPAN15* | rs59262400 | 0.300188 | *TSPAN15* |
| rs6030 | 0.240644 | *F5* | rs6050 | 0.217009 | *FGA* |
| rs6050 | 0.234128 | *FGA* | rs6030 | 0.208518 | *F5* |
| rs75112989 | 0.203980 | *ATP1B1* | rs75112989 | 0.196367 | *ATP1B1* |

*Changes to the manuscript, P 15 l 2:*

**Table 4F:** Table of the SNPs with the highest feature importance for the genetic, and mixed pneumonia linear models

| **Genetic, SNP** | **Coefficient (positive prediction)** | **Gene** | **Mixed, SNP** | **Coefficient (positive prediction)** | **Gene** |
| --- | --- | --- | --- | --- | --- |
| rs8062405 | 0.352614 | *ATXN2L* | rs10519203 | 0.353799 | *HYKK* |
| rs10519203 | 0.253258 | *HYKK* | rs4788102 | 0.311939 | *SH2B1* |
| rs7498665 | 0.190802 | *SH2B1* | rs72793809 | 0.187079 | *ATXN2L* |
| rs72793809 | 0.143266 | *ATXN2L* | rs7498665 | 0.152760 | *SH2B1* |
| rs8034191 | 0.133021 | *HYKK* | rs10747050 | 0.119137 | *GRIN1* |

*Changes to the manuscript, P 17 l 5:*

**Table 4A:** Table of the SNPs with the highest feature importance for the single nucleotide polymorphism (SNP), and combined atrial fibrillation GLN-models

| **Top SNP in SNP model** | **Coefficient (positive prediction)** | **Gene** | **Top SNP in combined model** | **Coefficient (positive prediction)** | **Gene** |
| --- | --- | --- | --- | --- | --- |
| rs17042171 | 0.001151 | Intergenic | rs9940321 | 0.0006 | *ZFHX3* |
| rs52511 | 0.000464 | Not in NCBI | rs883079 | 0.000575 | *TBX5* |
| rs13376333 | 0.000352 | *KCNN3* | rs4399218 | 0.000567 | *LINC01681* |
| rs6658392 | 0.000280 | *KCNN3* | rs1895596 | 0.000548 | *TBX5* |
| rs2106261 | 0.000245 | *ZFHX3* | rs2106261 | 0.000503 | *ZFHX3* |

*Changes to the manuscript, P 18 l 18:*

**Table 4C:** Table of the SNPs with the highest feature importance for the single nucleotide polymorphism (SNP), and combined venous thromboembolism GLN-models

| **Top SNP in SNP model** | **Coefficient (positive prediction)** | **Gene** | **Top SNP in combined model** | **Coefficient (positive prediction)** | **Gene** |
| --- | --- | --- | --- | --- | --- |
| rs8176745 | *0.000873* | *ABO* | rs505922 | *0.000388* | *ABO* |
| rs8176719 | *0.000679* | *ABO* | rs6050 | *0.000241* | *FGA* |
| rs10800454 | *0.000382* | *F5* | rs8176719 | *0.000234* | *ABO* |
| rs8106664 | *0.000381* | *SLC44A2* | rs8176745 | *0.000161* | *ABO* |
| rs10094510 | *0.000244* | *ZFPM2* | rs1593 | *0.000160* | *F11* |

*Changes to the manuscript, P 20 l 5:*

**Table 4E:** Table of the SNPs with the highest feature importance for the single nucleotide polymorphism (SNP), and combined pneumonia GLN-models

| **Top SNP in SNP model** | **Coefficient (positive prediction)** | **Gene** | **Top SNP in combined model** | **Coefficient (positive prediction)** | **Gene** |
| --- | --- | --- | --- | --- | --- |
| rs17851582 | 0.000663 | *GAMT* | rs9353801 | 0.000614 | Intergenic |
| rs2505594 | 0.000549 | Intergenic | rs12459346 | 0.000579 | Intergenic |
| rs10105477 | 0.000344 | Intergenic | rs7256201 | 0.000452 | *LOC107985327* |
| rs72793809 | 0.000309 | *ATXN2L* | rs72976957 | 0.000328 | *PIAS4* |
| rs4426179 | 0.0000243 | Intergenic | rs10503997 | 0.000324 | Intergenic |
